# Editorial

**Published:** 1861-09

**Authors:** 


### Editorial.

The Chicago Medical Journal and Lind University.—After three months delay, the June and July numbers of the *Chicago Medical Journal* (consolidated into one) have come to hand. In glancing over their pages, we find the following paragraph, which is not only inexcusably *mean* in its intended bearing on the University, but is also destitute of even the semblance of truth:

"Most of our readers may have heard of the Lind University, and the great medical reform department, established by Mr. Lind, with worthy co-operators. The following extracts from the Chicago *Daily Tribune*, of Thursday, June the 27th, 1861, *exhibit a few only of the facts which the public ought to know, concerning the source of the funds with which Mr. Lind may have been able to reform and convert the world medical and non-medical:* "It is only a matter of common fairness to state that the diversion and misappropriation (of the city funds) are laid solely to the act of *its* treasurer, Sylvester Lind." "It is not a pleasant reflection that a man who could endow a sectarian institution of learning to the amount of $100,000, should, by his official misconduct, cause the *indefinite suspension of our public schools.*"

A careful reading of this paragraph will show that it fairly implies the following propositions:

1st. That Sylvester Lind was the chief agent in establishing the Medical Department of the Lind University.

2d. That he endowed the University with $100,000, and derived the *means for doing it from the city funds*, entrusted to him as *its treasurer.*

3d. That such perversion of the public funds had caused the indefinite suspension of our public schools.

Were it not for those readers of medical journals abroad, it would be unnecessary to take any notice of statements which are so well known here to be entirely false in every particular. Mr. Lind had nothing to do with the establishment of the Medical Department of the Lind University, except to *rent* a part of a block of buildings for its occupancy, for which he was paid a fair rental annually. He never actually endowed any department of the University with a single dollar, and consequently, never used any city funds for that purpose. Years ago, before Mr. Lind had anything to do with the public funds of the city, he *offered* to endow the Theological Department of the University with $100,000, on certain conditions. Those conditions could not be complied with by the Board of Trustees, and consequently the offer was never carried into effect.

The third proposition, that Mr. Lind's default had caused the "indefinite suspension of our public schools," is too ridiculous, even for a sensation paragraph in a newspaper.

The truth is, Mr. Lind never had a dollar of the city school funds in his hands, or any funds that could in any way influence the public schools. He was simply Treasurer of the Board of Sewerage Commissioners, and all the money in his control was for *sewerage purposes*, and in nowise connected with schools, either public or private. All these facts were well known to the editors of the *Chicago Medical Journal*, when they inserted the above paragraph; and hence, they could have had no other motive than the hope of perverting the delinquencies of an individual to the discredit and injury of a public educational institution. The friends of the Lind University, and of the course of education generally, may rest assured that the failure of Mr. Sylvester Lind, does not in any degree, affect the prosperity of that or any other educational institution. The University and the public schools of the city, are as active, prosperous, and useful to-day, as at any time in their history.

Changes in Medical Colleges in this City.—The extensive military organizations and movements, going on in every part of the country, leave but few institutions unaffected. They have caused, at least, temporary changes in the faculties of both the medical colleges of this city. M. K. Taylor, M. D., Prof. of Pathology and Public Hy[illegible]ne, and H. Wardner, M. D., Demonstrator of Anatomy, ha[illegible] both accepted appointments, and are doing duty as surgeons in the volunteer regiments of this State. Their places in the Medical Department of the Lind University, have been *temporarily* filled in such a manner, as to prevent the omission of a single lecture from the regular course of instructions. The full course on Pathology will be given by the Professor of Practical Medicine, and J. S. Jewell, M. D., will fill the place of Dr. Wardner, as Demonstrator.

From the faculty of the Rush Medical College, we learn that J. V. Z. Blaney, M. D., Prof. of Chemistry, and J. W.

FREER, M. D., Prof. of Surgical Anatomy and Microscopy, have been appointed Brigade Surgeons, and E. POWELL, M. D., Demonstrator of Anatomy, has accepted the post of Regimental Surgeon, and is already on duty. Dr. Powell's place in the college will be temporarily supplied by I. P. LYNN, M. D., aided by the Professor of Anatomy.

What provisions will be made for the courses of Professors Blaney and Freer, we do not know, and we think the matter, at this date, Sept. 1, is not definitely determined.

PERSONAL.—J. H. DOUGLAS, M. D., editor of the *Am. Med. Monthly*, having been appointed on the *Sanitary Commission*, L. ELSBERG, M. D., editor of the foreign department of the *Monthly*, succeeds him in the control. Dr. E. is an impartial, able and scholarly writer, and the *American* will lose none of its well-earned reputation in his hands......Drs. PRINCE, FREER, BLANEY, RAUCH, HAVEN and SIM have been appointed Brigade Surgeons, in the Federal army, from this State. Dr. Blaney is assigned to General Hunter's Division, Drs. Freer, Sim and Rauch go to Washington, and the remainder are not yet assigned......Dr. W. H. DAVIS, of Paris, has been appointed Regimental Surgeon, and his place on the Board of Medical Examiners is filled by Dr. GREEN, of Salem. The Board still holds daily sessions in this city, but will adjourn to Springfield on the 16th. They have passed, since our last, eighteen additional surgeons and twenty-six assistant-surgeons—making in all, forty-two of the former, and fifty-one of the latter grade passed......Dr. R. M. HODGES, of Boston, a surgeon of promise, and a skilful anatomist, has received the Boylston Medical Prize for 1861, for the best dissertation on *Excision of the Joints*......HORACE G. SPAFFORD, Esq., Professor of Medical Jurisprudence in the Medical Department Lind University, was married in this city, on the 5th inst., to Miss ANNIE LARSON.

RENUNCIATIONS OF HOMŒOPATHY.—Under the head of selections in this number of the EXAMINER, will be found a letter from JOHN C. PETERS, M. D., of New York, for several years a prominent homœopathic practitioner and editor of the leading homœopathic journal in this country, formally renouncing all further connection with that pretended *system* of medicine. In the *American Medical Times*, for August 31st, we find a declaration, renouncing their further adhesion to homœopathy, signed by Ed. P. Fowler, M. D., Wm. Faulkner Browne, M. D., and Wm. O. McDonald, M. D., all heretofore well known as homœopathic practitioners in the city of New York. A similar declaration from most of the little-pill fraternity in this city, would bring their *professions* and *practice* more nearly into correspondence.

Further Experiments with Kerosolene.—In the absence of a report from the committee appointed at the July meeting of the Cook County Medical Society, to investigate the properties and actions of the new anæsthetic, kerosolene, it may be as well to put on record one or two of the experiments made with this agent on the lower animals, by the junior editor of the Examiner.

Expt. I. A full-grown, healthy doe rabbit was subjected to the influence of the vapor, administered on a cotton cloth. After inhaling about two minutes, during which the respiration became hurried and convulsive, the animal screamed loudly and continuously for more than a minute, followed by violent struggling, which, at the end of the fifth minute (the inhalation meanwhile continued), gave place to rapid involuntary motion of the fore-legs. The animal was now laid on its side, and the inhalation suspended, but the involuntary motion of the fore-legs was continued, the posterior extremities lying relaxed. At the end of the eighth minute (the second of the intermission), the involuntary motions had given way to attempts to regain its feet; breathing natural, though a little hurried, and vapor again exhibited. The struggling was at once renewed, followed by strong, clonic spasms of the extremities, varied by the rapid movements of the fore-paws, during which the hind-legs were rigidly extended; to this succeeded violent shivering of the whole body, respiration very quick and labored, eye-balls protruded. At the end of the twelfth minute, sensibility was yet perfect, the prick of a scalpel being instantly responded to. The animal now lying prone, the cloth was placed close under its nostrils, and two drachms of the fluid poured on, care being taken that it did not touch the nostrils. The irregular convulsive movements were again renewed, intermitting to fits of shivering, after one of which the trunk was flexed backwards (opisthotonos), extremities extended and respiration ceased. On opening the thorax, ten minutes afterwards, the right auricle was found immensely distended, and still strongly pulsating, pulmonary arteries congested, and the other usual symptoms of asphyxia. The inhalation was continued nearly fifteen minutes, with about two minutes intermission, and at no time was there any anæsthesia.

Expt. II. A full-grown, young male rabbit was subjected to the vapor, by pouring half an ounce of the liquid into a common tumbler, into the upper part of which pussy's head was confined. For the first five minutes the phenomena were similar to those in the first experiment; but while struggling, his head was so far released as to allow him to lap up, probably, half a drachm of the fluid. Violent shivering fits at once supervened, followed by entire muscular relaxation, from which (the vapor being withdrawn), he recovered in four or five minutes, eating and running with perfect freedom. No anæsthesia.

Expt. III. The same rabbit, one week afterwards, was again treated to the vapor from the tumbler. Phenomena almost identical with those of No. I., death occurring in about fourteen minutes, with no intermission of the supply of vapor. Post-mortem revealed the usual symptoms of asphyxia. The blood coagulated very slowly in both instances. Ten drachms of kerosolene were used in the first, and not quite an ounce in the last experiment. Anæsthesia was not produced in either case, at any time before death.

---

Military Hospital Treatment of Gonorrhœa.—The statement that "venereal cases constitute at least, one-third of the admissions into military hospitals," will, undoubtedly, suffer a large discount, when applied to the volunteer service,—for obvious reasons. And yet the frequent occurrence of "the transitory penalty of social indiscretion" among our troops, and the consequent annoyance, to say nothing of the disastrous possibilities, of crowded hospitals and weakened regiments, renders pertinent and valuable, at this time, the publication of any approved plan of treatment, even though obnoxious to the charge of gratifying "an unwholesome love of novelty."

Assistant-Surgeon Miles, M. R. C. S. L., etc., in Medical Charge, at Halifax, N. S., contributes a paper to the current No., *London Lancet*, detailing a plan of treatment, whose success he has abundantly tested for eighteen months, and in some sixty cases; and for which he claims that, whilst it is scarcely open to an objection, it offers at once a speedy and effectual mode of cure, returns the soldier's name to the list of "effectives for duty," in the shortest practicable time, is the best suited for soldiers, and is least open to tampering and deceit. After establishing a soluble condition of the bowels, in a case marked by the usual symptoms: penis swollen and glans cherry-colored, tender, and looking excoriated; lips of orifice of urethra puffy; with an abundant, thick, slightly yellowish discharge; urine scanty, and the stream forked and painful in its passage, a blister, six inches by four, is ordered to be placed very high up towards the anterior and inner aspect of each thigh. The blisters are of the London Pharmacopœia, the plaster being spread rather thickly, and the surface afterwards sprinkled with acetate of cantharides, a wide margin being left on each side, of the blister. These are safely confined in place by a broad strip of adhesive plaster at each end. For convenience the blisters are generally directed to be applied at night, and no harm has ever come of it, and usually they are well risen by the morning. The patient is then ordered to take every four hours an ounce of the following mixture:—sulphate of magnesia, two ounces; carbonate of magnesia, four drachms; potassio-tartrate of antimony, two grains; tincture of hyoscyamus, two drachms; peppermint water, sixteen ounces. He is placed on spoon diet, with rice pudding for dinner, and a pint of imperial drink should he be thirsty. During the day he is directed to inject now and then a syringeful of cold or lukewarm water, according to the temperature and season of the year. The blistered surface to be dressed with lint dipped in castor oil (a favorite dressing with the men).

The immediate effect is not uniform,—in some cases the discharge is increased, with more scalding; in others, discharge is less, but thicker; in still others it ceases at once; the usual result is an aggravation of the symptoms for the first twenty-four hours; the only thing required, however, is a little patience combined with perfect rest, and these symptoms rapidly subside. The saline purgative is to be continued, and the patient is ordered half an ounce of gum arabic to be mixed with water, and used as a drink, whenever required, the imperial drink being continued if asked for. On the third morning after the application of the blisters, the discharge is noticed to be much diminished in quantity, the scalding in micturition is less, and the patient feels better. On the fourth day scarcely any discharge, and there is now no scalding. The blistered surfaces look raw, but are beginning to "skin," as the men say. The saline purgative to be now given only three times a day. The next day perhaps there is a little running when the penis is squeezed, but no other symptom; his bowels are well open. To take the saline purgative (two ounces) every morning, and an injection of nitrate of silver, (six grains to the ounce) to be used at night. Sixth day: Blistered places skinned, though not firmly, and the surfaces are not sore. No sign of running is visible to-day, but there is an appearance as if of weeping at the lips of the urethra. Seventh day: No sign of discharge, the thighs have completely cicatrized, and the surface where they were blistered, is not the least tender. The man is discharged for duty, with one day's convalescent leave. This, though a suppositious case, illustrates the sort of treatment adopted. But a more favorable case than the above will happen. A lad may come the morning after the discharge appears, and in the early stage of the complaint very mild inflammatory symptoms are present. He is of temperate habits. In such a case, all that may be required are, rest and low diet, the saline purgative mixture, and as a local remedy, a small blister applied to the under surface of the penis. The blister should be about an inch wide, and from an inch to an inch and a half long, and be carefully fastened on by oiled threads. It is well to fasten a suspensory bandage, with some extra linen covering over the scrotum, so as to prevent the chance of vesicating it. If the blister rises properly, in a simple case of gonorrhœa taken thus early, I have had repeated instances of immediate cure by a single application, the discharge having completely ceased, with no recurrence at a subsequent period. In such a case, the blistered surface has healed (it is surprising the rapidity with which it does so on this spot), and the man has been discharged for duty on the fourth morning after admission.

The Surgeons of the New York Eighth.—The Washington correspondent of the N. Y. *Times*—a secular paper, which, alone of the hundreds published in the United States, gives to the profession a courteous attention and consideration,—speaks in the following glowing terms of the conduct of Surgeons Foster Swift and Charles De Graw, of the Eighth Regiment N. Y. S. M., at the battle of Bull Run. What these gentlemen did was no more than their duty, to be sure, and no more than did Buckstone, Allen and Williams, of Maine, Norvell, Powell, Winston, Griswold, Peugnet, Ferguson, Harris, Homeston and Swalm, of New York, Taylor, of New Jersey, Lewis, of Wisconsin, Stewart, of Minnesota, and Steinberg and Grey, of the regular army, on the same occasion; and no more, we firmly believe, than every medical man—who goes forth from the comforts and emoluments of a civil practice, with its unquestioned position and privileges, to the hardships, dangers and exigencies, the petty pay, the dubious rank and limited authority of military service,—will be ready and prompt to do. The quality of heroism demanded for such service must be higher than that of the mere combatant;—since to it there are none of the usual stimuli which attend martial life, robbing privation, and fatigue and danger of their worst features. The wild enthusiasm of the battle-shock must be sternly repressed by the surgeon who would do his duty calmly and fully; danger, suffering and death, in their most terrible and repulsive forms surround him; yet, his heart must not shrink, nor his nerve falter. No special mention is made in "Official Reports,"—no brevets on the field for meritorious conduct,—no incentive of promotion to nerve and sustain him. Are we not justified, then, in reprinting such unusual recognition of the usual conduct of our professional brethren?:—

We have enough of buncombe receptions, but of real approbation of true heroism, how much do we see?

Two men return to New York to-night who are, in the truest use of the word, heroes. These are the surgeons of the Eighth New York Regiment, whose officers deserted in many cases before coming under fire. They were in the Sudley Church; they arrived at it amid the hottest rush of the mad panic, but stopped to aid in taking care of the wounded, who had been placed in it. They were warned that the enemy were approaching; they heard the cry that the enemy were bayoneting the wounded, and giving no quarter; they were advised to leave by a United States officer, who told them the enemy would undoubtedly shell the church on the supposition that it would be held as a defensive position as long as possible, by our troops covering the retreat. This conviction was so strong with those who left last, as you will remember, that it was generally reported the church was shelled. But these surgeons said, simply, "*We cannot leave these bleeding men; it is our business to take care of them, and take care of them we must, no matter what becomes of us.*" They unquestionabiy made up their minds to die, and momentarily expected the falling of the first shell among them, until, while in the midst of an amputation, there was a rush of cavalry, and a horseman's pistol was pushed in at the door, and a hoarse voice asked, "Do you surrender?"

"Certainly," answered Swift, tying an artery, and not so much as raising his head from his business.

Would to God we had a few score such men as captains, and would to God we had a general who would blow from the cannon's mouth such sham officers as those who played soldier with the Fire Zouaves and the Eighth.

Successful Operation for Double Congenital Cataract at the Age of Eighteen.—Dr. Welsh translates for the *Boston Med. and Surg. Journal*, the correspondence of A. M. Cade, M. D., with the *Bull. Gén. de Thérap. Méd.*, in which he details a successful operation for double congenital cataract, of eighteen years duration. The operation was by depresso-reclination, as M. Cade terms a mixed procedure, by which, before tipping the lens into the inferior and external part of the vitreous humor, it is depressed vertically until a semilunar opening appears in the upper fifth of the pupil; thus preventing, during the displacement of the cataract, both the falling of the lens into the anterior chambers, and the too immediate compression of the retina or choroid coat. On the eighth day the left eye was completely cured, and the right only occasionally obstructed by the still floating unabsorbed cataract. This, however, would doubtless be promptly absorbed.

In addition to the rarity of operations for congenital cataract after the age of puberty, we quote the case partially for the interesting phenomena resultant on the sudden acquisition of vision after eighteen years blindness. M. Cade says:

The first sensation of light produced so lively a sensation, that the eyes were seized with convulsive movements, and it was only after having established twilight in the room, and after numerous oscillations, that the globes of the eyes maintained their equilibrium. When the organs of vision were accustomed to the light, I attempted some experiments and observed the following facts: when an object is presented to Mdlle. Louise, she can neither appreciate its form or color; she is obliged to touch it, to tell its name or use. The laws of visual accommodation are lost for her; thus, she judges distances so inaccurately, that she constantly places her hand beyond the objects she wishes to grasp. Besides, this young lady has been so in the habit of using the sense of touch to supply that of sight, that, after having the name of an object pointed out to her, she feels the need of taking hold of it and manipulating it in every direction, so as to fix the form of it as well as its other characteristics in her memory. When an object has been presented to this double inspection, it remains impressed on the memory, and Mdlle. Louise can name it by the exclusive use of her eyes, even four or five days after a first trial.

